# *In-vitro* selection of lactic acid bacteria to combat *Salmonella enterica* and *Campylobacter jejuni* in broiler chickens

**DOI:** 10.1007/s11274-024-03946-8

**Published:** 2024-03-14

**Authors:** Ramesha N. Wishna-Kadawarage, Rita M. Hickey, Maria Siwek

**Affiliations:** 1 Department of Animal Biotechnology and Genetics, Faculty of Animal Breeding and Biology, Bydgoszcz University of Science and Technology, Mazowiecka 28, Bydgoszcz, 85-084 Poland; 2 Teagasc Food Research Centre, Moorepark, Fermoy, Co. Cork, P61 C996 Ireland

**Keywords:** Anti-*Campylobacter*, Anti-*Salmonella*, Foodborne pathogens and organic acids

## Abstract

*Campylobacter* and *Salmonella* are the two most prominent foodborne zoonotic pathogens reported in the European Union. As poultry is one of the major sources of these pathogens, it is imperative to mitigate the colonization of these pathogens in poultry. Many strains of lactic acid bacteria (LAB) have demonstrated anti-*Salmonella* and anti-*Campylobacter* characteristics to varying degrees and spectrums which are attributed to the production of various metabolites. However, the production of these compounds and consequent antimicrobial properties are highly strain dependent. Therefore, the current study was performed to select a potent LAB and determine its causal attribute in inhibiting *Salmonella enterica* and *Campylobacter jejuni*, *in-vitro*. Six LAB (*Lactiplantibacillus plantarum* (LP), *Lacticaseibacillus casei* (LC), *Limosilactobacillus reuteri* (LR), *Lacticaseibacillus rhamnosus* (LRh), *Leuconostoc mesenteroides* (LM) and *Pediococcus pentosaceus* (PP)) and three serovars of *Salmonella enterica* (Typhimurium, Enterica and Braenderup) and *Campylobacter jejuni* were used in the current study. Spot overlays, well diffusion, co-culture and co-aggregation assays against *Salmonella* and well diffusion assays against *Campylobacter jejuni* were performed. Organic acid profiling of culture supernatants was performed using HPLC. The results indicated that LRh, LM and PP had the most significant anti-*Salmonella* effects while LP, LC, LM and PP displayed the most significant anti-*Campylobacter* effects. Lactic acid and formic acid detected in the culture supernatants seem the most likely source of the anti-*Salmonella* and anti-*Campylobacter* effects exhibited by these LAB. In conclusion, *Leuconostoc mesenteroides* displayed the most significant overall anti-pathogenic effects when compared to the other LAB strains studied, indicating its potential application *in-vivo*.

## Introduction

Foodborne pathogens are the microorganisms which may transmit to humans via consumption of certain foods (Bintsis 2017). According to the latest reports, *Campylobacter* and *Salmonella* are the two most prominent foodborne zoonotic pathogens reported within the European Union (Authority EFS. & European Centre for Disease Prevention and Control 2022). *Salmonella* is also known as the foodborne pathogen with the highest number of reported human hospitalizations in the United States (Centers for Disease Control and Prevention 2022). Approximately, one million people become sick in the United States each year due to consumption of contaminated poultry products and the Center for Disease Control claims that chicken is one of the major sources of *Salmonella* and *Campylobacter* pathogens in humans. On the other hand, as per the estimations published by European Food Safety Association (EFSA) in 2020 updating the 2011 opinion, a 10^3^ reduction of *Campylobacter* contamination in chicken ceca can cause a 58% reduction of the public health risk (Hazards (BIOHAZ) et al. 2020). Therefore, it is imperative to find solutions to mitigate *Salmonella* and *Campylobacter* prevalence in broiler chickens to combat foodborne infections and assure food safety worldwide.

Lactic acid bacteria (LAB) have been intensively studied over the past few decades with the aim of harnessing their antimicrobial properties as alternatives to antibiotics in livestock production. Consequently, many LAB strains have been shown to possess anti-pathogenic effects against *Salmonella* and *Campylobacter* and have been used in the food industry due to their antimicrobial food preservation abilities (Reviewed by Vieco-Saiz et al. 2019 and Ibrahim et al. 2021). Furthermore, many LAB strains are identified by the Food and Drug Administration (FDA) under the status of Generally Recognized As Safe (GRAS) and by EFSA under the status of Qualified Presumption of Safety (QPS) and as such, have been used in the food and feed industry for many years (Webb et al. 2022). LAB consist of diverse genera of bacteria which produce different metabolites or compounds which possess antimicrobial properties. Bacteriocins are the one type of antimicrobial compound that are known to be produced by some of the LAB strains. These are antimicrobial peptides with either a broad or narrow spectrum of antimicrobial ability (Wyszyńska and Godlewska 2021). Their mechanisms include disruption of cell wall synthesis and pore formation in cell wall/membrane of pathogens inhibiting their growth and survival (Kumariya et al. 2019). Another important attribute of LAB associated with anti-pathogenic properties, is the production of organic acids (Cizeikiene et al. 2013). Among these organic acids, lactic acid, acetic acid and formic acid, are the major by-products of LAB that are associated with a broad spectrum anti-pathogenic effects. These organic acids create a low intracellular pH environment where pathogens cannot perform their regular metabolic functions such as replication and protein synthesis (Vieco-Saiz et al. 2019). Apart from bacteriocins and organic acids, some LAB can produce hydrogen peroxide, diacetyl, ethanol and carbon dioxide also providing antimicrobial activity against wide range of pathogens (Vieco-Saiz et al. 2019; Wyszyńska and Godlewska 2021; Webb et al. 2022).

Considering the potential of LAB to produce such antimicrobial metabolites against pathogenic bacteria, we selected a number of LAB to screen for the strain with the most broad spectrum of activity in inhibiting different strains of *Salmonella* and *Campylobacter jejuni* in broiler chickens. However, the antimicrobial characteristics are highly dependent both on the probiotic and pathogenic strains chosen (Campana et al. 2017). Therefore, *in-vitro* selection of LAB strains for antimicrobial applications in livestock production required specific focus on certain LAB strains. Accordingly, six commercial LAB strains (homofermentative, obligatory heterofermentative and facultative heterofermentative) belonging to different genera, were chosen for screening against strains of *Salmonella enterica* and *Campylobacter jejuni* under *in-vitro* conditions.

## Materials and methods

### Bacterial strains

Six LAB strains (which are currently commercially used in multi-strain probiotic supplements for swine and poultry and produced by JHJ Sp Z.o.o, Nowa Wieś, Poland) were selected for anti-pathogenic screening. All the LAB strains had been identified using 16s rRNA sequencing and deposited at the Polish collection of Microorganisms located in Wrocław. The pathogens used in the study included three serovars of *Salmonella enterica* subspecies Enterica and one strain of *Campylobacter jejuni* (Table 1).

**Table 1:** Lactic acid bacteria and pathogenic strains used

LAB		Pathogens	
Strain	Origin	Strain	Origin
*Lactiplantibacillus plantarum* B/00166 (LP)	Swine	*Salmonella enterica* subsp. Enterica serovar Typhimurium (DPC6463)	Chicken
*Lacticaseibacillus casei* B/00164 (LC)		*Salmonella enterica* subsp. Enterica serovar Typhimurium (ATCC 14028)	
*Limosilactobacillus reuteri* B/00281 (LR)		*Salmonella enterica* subsp. Enterica serovar Braenderup (NRL-IE-22)	
*Lacticaseibacillus rhamnosus* B/00279 (LRh)		*Campylobacter jejuni* DVI-SC181	
*Leuconostoc mesenteroides* B/00288 (LM)			
*Pediococcus pentosaceus* B/00165 (PP)	Chicken		

### Anti-*Salmonella* assays

#### Spot overlay assays

LAB were inoculated into MRS broth (BD 288130) and incubated aerobically at 37^°^C for 20 h. Five microliters of each LAB culture were spotted into a labelled MRS agar plate allowed to air dry. These plates were incubated at 37^°^C overnight. Fifteen microliters of cultures of each *Salmonella* strain (incubated at 37^°^C for 16 h in BHI broth (1.10493 Merck)) was added to 30 ml of BHI molten cooled (at 50^°^C) agar (0.75%) and mixed gently. The *Salmonella* inoculated agar was overlaid the plate containing LAB spots grown overnight and was further incubated at 37^°^C overnight. The zone of inhibition surrounding the LAB spots were measured in mm (Four measurements of the radius were taken perpendicularly and averaged). The experiment was performed in triplicate. The three most promising LAB which displayed highest inhibition of all three *Salmonella* strains were selected for further assays.

#### Well diffusion assays (WDAs) against *Salmonella* Typhimurium

The overnight cultures of the selected strains were prepared as described in spot overlay assays section. These cultures were centrifuged at 4000 g for 15 min at 4^°^C and the supernatant was retained. The pH of the cultures (grown for 20 h) was determined using a pH meter. Supernatant obtained from each culture was neutralized using 1 M NaOH or 1 M HCL, to pH 7 ± 0.2. Untreated and pH neutralized supernantants were filter sterilized using 0.22 μm syringe filters.

*Salmonella* Typhimurium (DPC6463) overnight culture was prepared as described in spot overlay assays section and 25 µl of the culture was inoculated in 50 ml of BHI molten cooled (at 50^°^C) agar (1%) and was mixed gently. The inoculated molten agar was poured into a square petri dish and allowed to set for 20 min. Wells of approximately 7 mm in diameter were created in the inoculated agar aseptically, using a sterile pipette tip (1000 µl). Each well was labelled with the names of LAB and 100 µl of the filtered LAB culture supernatants (neat and pH neutralized) was added into the respective wells. For the WDA with neat LAB supernatants, MRS broth (pH = 4) was used as a negative control. The wells were dried at room temperature in a laminar flow hood to the point that when moved to the incubator, the liquid in the wells was not displaced (approximately 30 min). Then the plates were incubated at 37^°^C for 16 h. Inhibition around the wells were observed and recorded (in mm). The experiment was performed in triplicate.

#### Co-culture assays

The three LAB which exhibited the strongest inhibition of all three *Salmonella* strains were selected for co-culture experiments. Double strength BHI broth (for *Salmonella*) and MRS broth (for LAB) were prepared. Double strength MRS was mixed in equal volume with double strength BHI for the co-culture experiment of LAB with *Salmonella*. The mixture of double strength media (10 ml) was inoculated with 100 µl of each LAB overnight culture (incubated for 20 h) and 100 µl of *Salmonella* Typhimurium culture (incubated for 16 h) and incubated for 24 h at 37^°^C. Selective enumeration of *Salmonella* Typhimurium in each coculture was performed at 0, 5, 10 and 24 h time points using spot plate method on *Salmonella* chromogen selective agar (CM1007). Results were graphed to visualize the growth of *Salmonella* in presence and absence of LAB. The experiment was performed in triplicate. The pH of the cultures was also recorded at each time point.

#### Co-aggregation assay with *Salmonella* Typhimurium

The co-aggregation ability of a bacterium is an indicator of the potential inhibition of the colonization of a pathogen in the gut by a beneficial bacteria which co-aggregates with it. Therefore, the co-aggregation ability of the three LAB selected was tested together with *Salmonella* Typhimurium. All bacterial overnight cultures were prepared as described in the spot overlay assays section. Cultures were centrifuged at 4000 g for 15 min at 4^°^C. The supernatant was discarded and cell pellet was washed with sterile PBS twice. Then the cell pellet was re-suspended in PBS to a concentration of 0.5 optical density at 600 nm (OD_600_). OD_600_ measurements were obtained using BioTek Synergy HT microplate reader. Five hundred microliters of each bacterial suspension was aliquoted into a sterile flat bottom 48 well microtiter plate. Additionally, 250 µl of each LAB suspension was added with 250 µl of *Salmonella* suspension into the wells of the same plate and mixed by pipetting. The plate was then incubated at 37^°^C for 24 h. The OD_600_ reading of the wells was recorded using the microplate reader without shaking the plate. These experiments were performed in triplicate. The co-aggregation ability of each LAB was determined using the following formula (Balakrishna 2013).

$$\eqalign{ {\rm{Co}} & - {\rm{aggregation}}\;{\rm{ability}} \cr & = \left[ {1 - \left( {\left( {2 \times {\rm{Am}}} \right) \div \left( {{\rm{Al}} + {\rm{As}}} \right)} \right)} \right] \cr & \times 100 \cr}$$

Where;

Am = OD_600_ of mixture of LAB and *Salmonella* suspensions.

Al = OD_600_ of LAB suspension alone.

As = OD_600_ of *Salmonella* suspension alone.

### Anti-*Campylobacter* assays

#### Well diffusion assays against *Campylobacter jejuni*.

*Campylobacter jejuni* was inoculated in Mueller Hinton broth (BD 275730) supplemented with *Campylobacter* selective supplement (Skirrow) (SR0069E) according the manufacturer’s directions. After incubating the inoculated broth at 42^°^C for 48 h under microaerophilic conditions (5% O_2_, 10% CO_2_ and 85% N_2_) using CampyGen™ 2.5 L Sachet (CN0025A, Oxoid), Mueller Hinton agar (1.5%) plates (90 mm circular plates) were spread with 100 µl of this culture and were allowed to dry. Then, using a sterile 200 µl pipette tip, wells of approximately 5 mm in diameter were created aseptically in the agar. The LAB culture supernatants (both neat and pH neutralized) were added to each well (50 µl/well) and then the plates were left for approximately 30 min until the supernatants were absorbed into agar (wells were empty). These plates were incubated at 42^°^C for 24 h under microaerophilic conditions for 24 h. The inhibition zone around the wells was observed and recorded (in mm). The experiment was performed in triplicate.

### Organic acids characterization in culture supernatants

The culture supernatants (after 18 h of incubation) were filtered using 0.22 μm syringe filters. Levels of organic acid metabolites were then quantified by HPLC using a Waters Alliance Separations module e2695 coupled to a Waters 2414 refractive index (RI) detector (Waters, Milford MA, USA). Samples or standards at a volume of 20 µl were injected on to a Rezex Organic acids H + column (300 × 7.8 mm) operated at 60^°^C. The samples were eluted with H_2_SO_4_ (0.005 N) at a flow rate of 0.6 mL/min. Sample detection was performed by comparing retention times of standards. Analytical grade acetic acid, butyric acid, citric acid, lactic acid, formic acid and propionic acid supplied by Merck were used as standards. The assay was performed in duplicate.

### Statistical analysis of the data

The measurements from triplicate assays were used to perform ANOVA followed by Tukey’s HSD mean comparison test using Statistica software (Version 14.0.0.15) to identify statistically significant differences among the means.

## Results

### Anti-*Salmonella*

#### Spot overlay assays

**Fig. 1:**
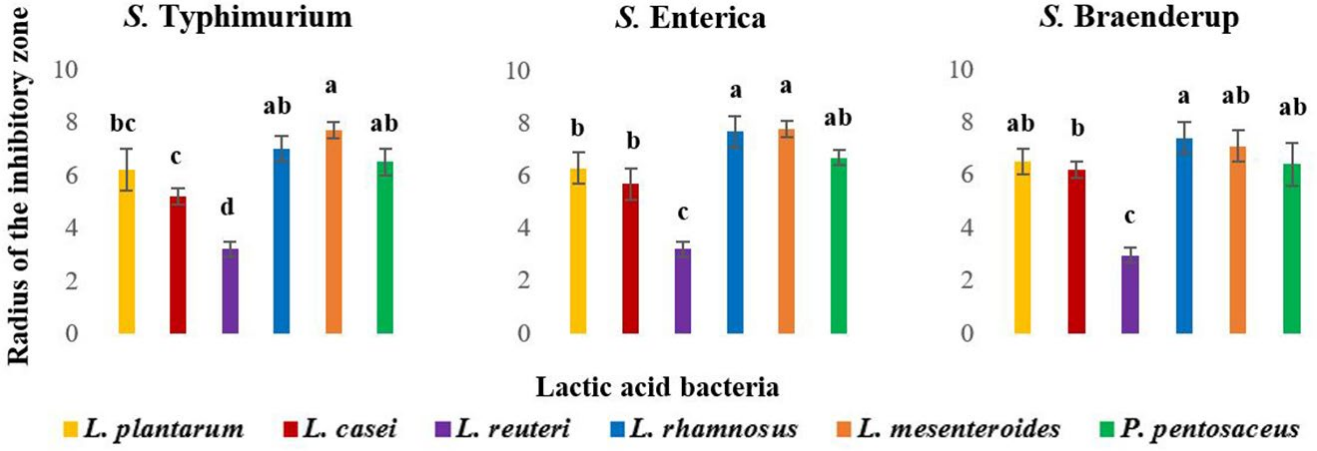
Radius of inhibitory zone (mm) observed in spot overlay assays against three *Salmonella enterica* serovars. Error bars: ±SD. Homogenous means have been indicated by similar letters identified by Tukey’s HSD test (p value < 0.05)

The results of the spot overlay assays indicated that five out of six LAB strains studied (except *L. reuteri*) are more effective against all three *Salmonella* serovars (Fig. 1). The highest overall anti-*Salmonella* activity was observed for *L. rhamnosus, L. mesenteroides* and *P. pentosaceus*. Therefore, these three LAB were used for further anti-*Salmonella* assays.

#### Well diffusion assays (WDAs)

**Fig. 2:**
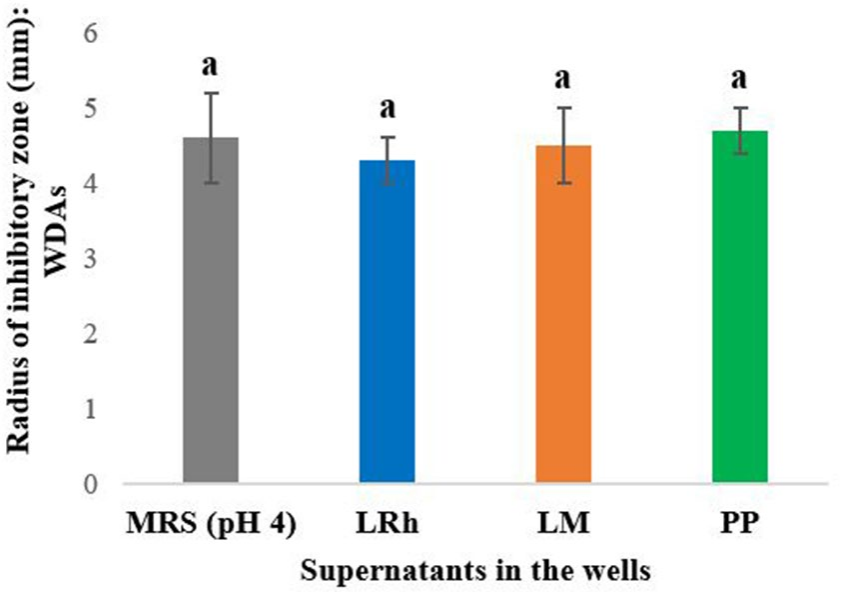
Radius of inhibitory zone (mm) observed in well diffusion assays (with neat supernatants) against *Salmonella* Typhimurium. **LRh**: *L. rhamnosus*, **LM**: *L. mesenteroides*, **PP**: *P. pentosaceus* Error bars: ±SD. Homogenous means have been indicated by similar letters identified by Tukey’s HSD test (p value < 0.05)

The pH of the culture supernatants obtained from the six LAB was approximately 4 (*L. plantarum-* 3.9, *L. casei-* 3.9, *L. reuteri- 4, L*. *rhamnosus*- 4, *L. mesenteroides-* 4.1 and *P. pentosaceus-* 4). In order to determine whether the inhibition observed by LAB in spot overlays was due to pH effect (via organic acid production), the WDAs against *Salmonella* Typhimurium were performed with neat (un-treated) and pH neutralized (pH 7 ± 0.2) culture supernatants of the three LAB selected. Interestingly, no inhibition was observed with the LAB supernatants when pH was neutralized indicating that anti*-Salmonella* effects observed are possibly due to pH effect/action of organic acids produced by the LAB. The neat supernatants however, displayed inhibition of *Salmonella* Typhimurium similar to MRS broth at pH 4 (Fig. 2). Therefore, it can be suggested that the.

#### Co-culture assays of LAB with *Salmonella*

**Fig. 3:**
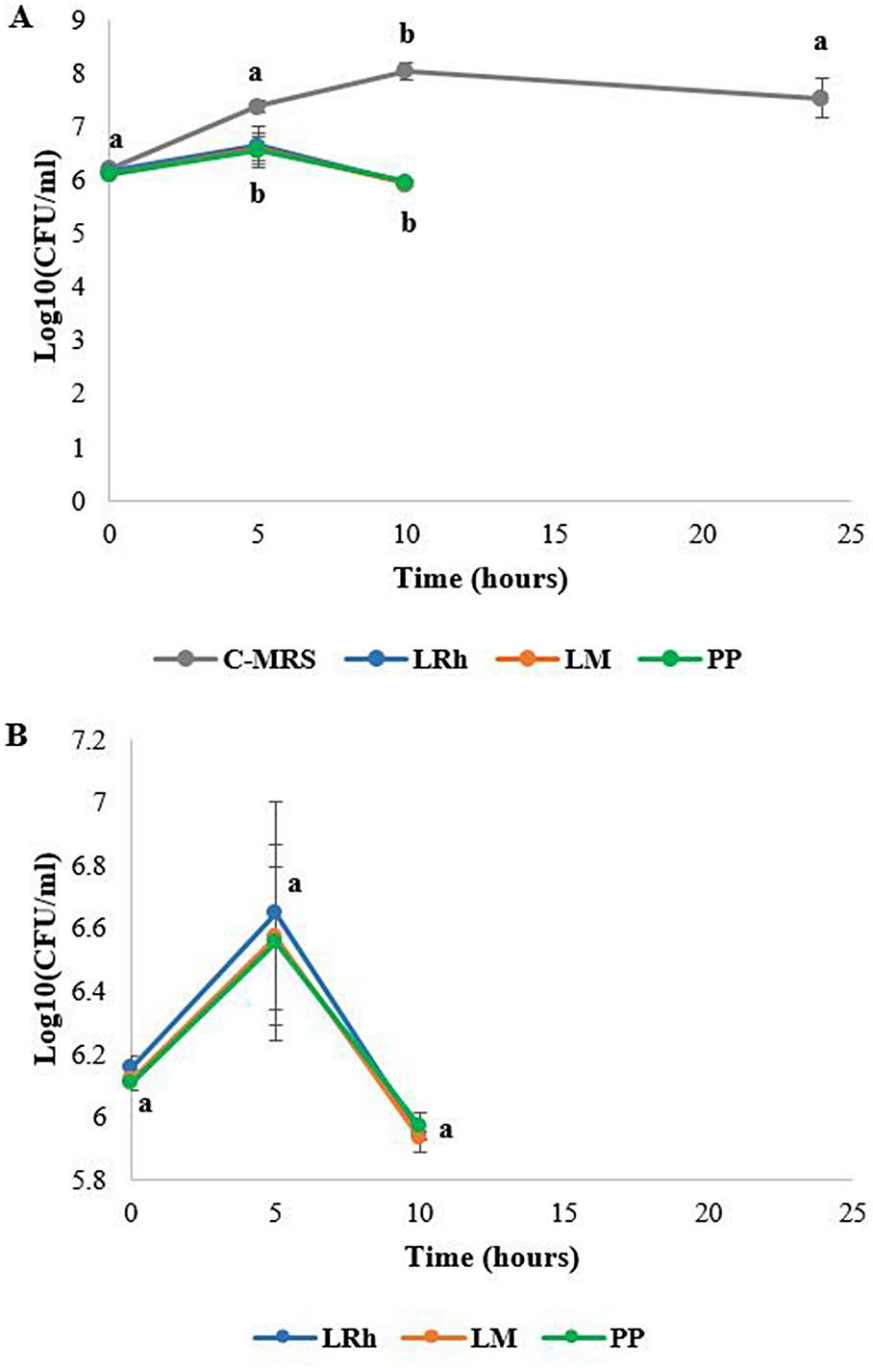
Selective enumeration of *Salmonella* Typhimurium in co-culture. **A**: Comparison of growth of *Salmonella* with and without LABs. **B**: Comparison of growth of *Salmonella* in co-culture with different LAB. **C-MRS**: Control media (MRS + BHI), **LRh**: *L. rhamnosus*, **LM**: *L. mesenteroides*, **PP**: *P. pentosaceus*. Error bars: ±SD. Homogenous means indicated by similar letters: Tukey’s HSD test (p value < 0.05)

The results of co-culture assay indicated that the three LAB strains selected (*L. rhamnosus, L. mesenteroides* and *P. pentosaceus*) based on promising inhibition observed with spot overlay assays, are equally efficient in inhibiting *Salmonella* Typhimurium. The number of colony forming units (CFUs) of *Salmonella* Typhimurium observed for in the presence of LAB was significantly lower when compared to the number of CFUs in the control medium (Fig. 3). Intriguingly, no colonies of *Salmonella* Typhimurium were present after plating the co-culture at 24 h indicating a complete eradication of *Salmonella* Typhimurium by LAB. These results suggest that the selected LAB strains possess both bacteriostatic and bactericidal properties against *Salmonella* Typhimurium.

**Fig. 4:**
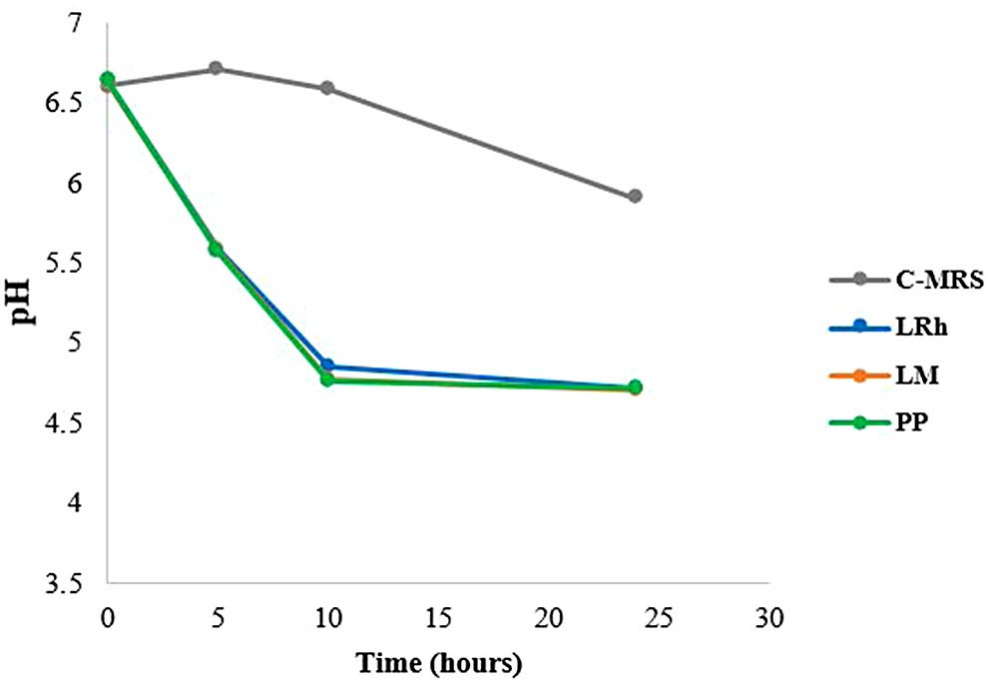
Changes of pH in the cultures of co-culture assay. **C-MRS**: MRS + BHI media control, **LRh**: *L. rhamnosus*, **LM**: *L. mesenteroides*, **PP**: *P. pentosaceus*

The pH of the co-cultures was measured over time (Fig. 4). It was observed that the pH of C-MRS (double strength BHI + MRS media) inoculated only with *Salmonella* Typhimurium gradually dropped to approximately 6 at the end of 24 h of culturing. However, co-culture with LAB strains decreased the pH to approximately 4.7 within first 10 h and remained constant until the end of 24 h. This result also supports the assumed role of organic acids produced by LAB in bactericidal effects on *Salmonella* Typhimurium.

#### Co-aggregation assays of LAB with *Salmonella*

**Fig. 5:**
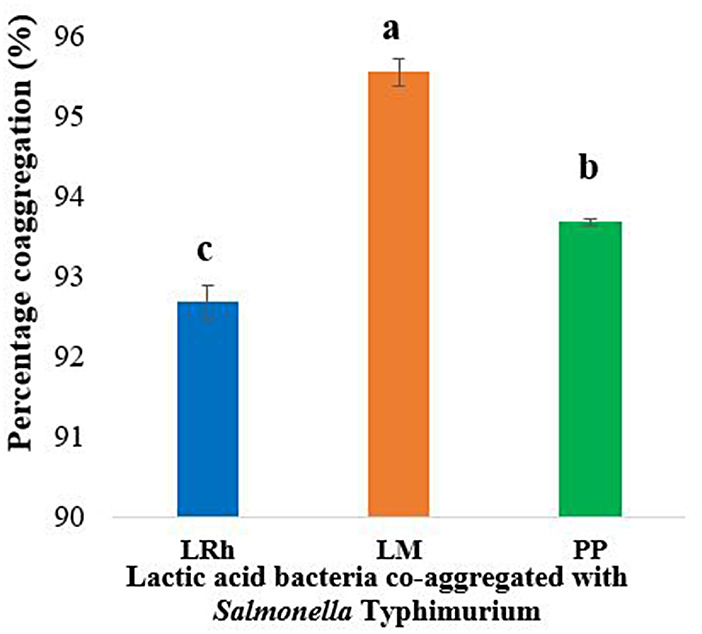
Results of co-aggregation assays of selected LAB strains with *Salmonella* Typhimurium. **LRh**: *L. rhamnosus*, **LM**: *L. mesenteroides*, **PP**: *P. pentosaceus.* Error bars: ±SD. Homogenous means indicated by similar letters: Tukey’s HSD test (p value < 0.05)

The co-aggregation assay was performed with the three most promising LAB strains (*L. rhamnosus, L. mesenteroides* and *P. pentosaceus*) together with *Salmonella* Typhimurium. The results (Fig. 5) indicated that highest co-aggregation is observed with *L. mesenteroides*.

#### Anti-*Campylobacter* well diffusion assays (WDAs)

**Fig. 6:**
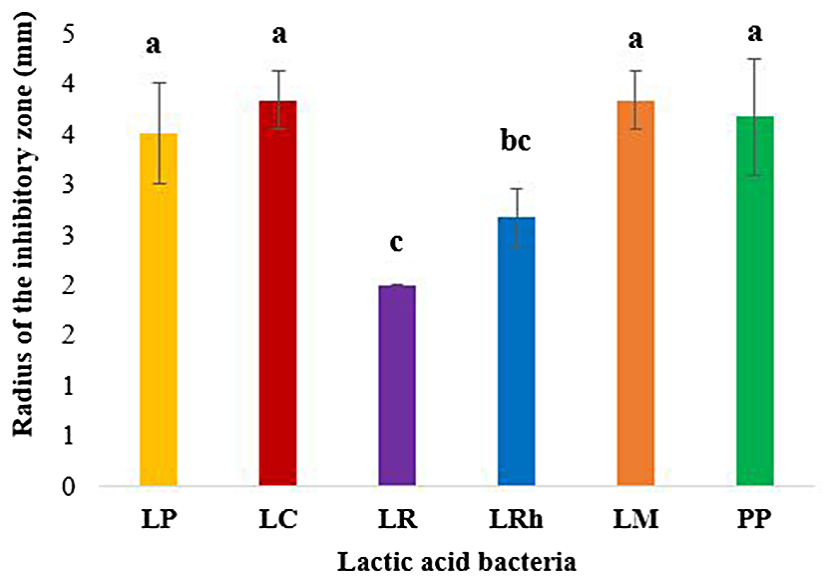
Inhibition of *Campylobacter jejuni* by neat culture supernatants in Well diffusion assays. **LP**: *L. plantarum*, **LC**: *L. casei*, **LR**: *L. reuteri*, **LRh**: *L. rhamnosus*, **LM**: *L. mesenteroides*, **PP**: *P. pentosaceus*. Error bars: ±SD. Homogenous means indicated by similar letters: Tukey’s HSD test (p value < 0.05)

**Fig. 7:**
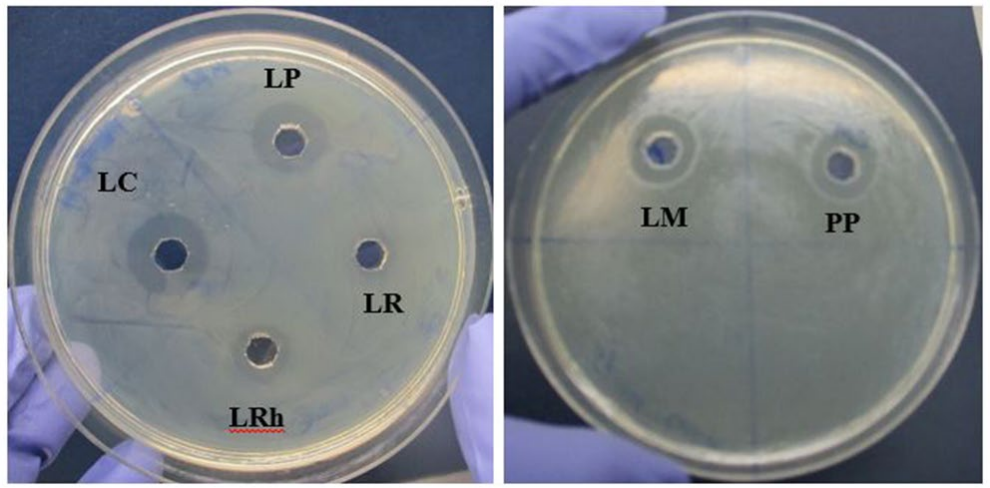
Anti-*Campylobacter* WDA results for culture supernatants (neat). **PC**: Positive control, **LP**: *L. plantarum*, **LC**: *L. casei*, **LR**: *L. reuteri*, **LRh**: *L. rhamnosus*, **LM**: *L. mesenteroides*, **PP**: *P. pentosaceus*

WDAs against *Campylobacter* was performed with LAB culture supernatants (neat and pH neutralized). The results indicated that *L. mesenteroides*, *P. pentosaceus* and *L. casei*, followed by *L. plantarum* displayed the highest inhibition of *Campylobacter jejuni* (Fig. 6). Similar to anti-*Salmonella* WDAs, no inhibition was observed with pH neutralized supernatants as opposed to the clear inhibitions observed with neat supernatants (Fig. 7) indicating a potential role of organic acids in anti-*Campylobacter* activity also.

### Organic acid characterization in culture supernatants

**Fig. 8:**
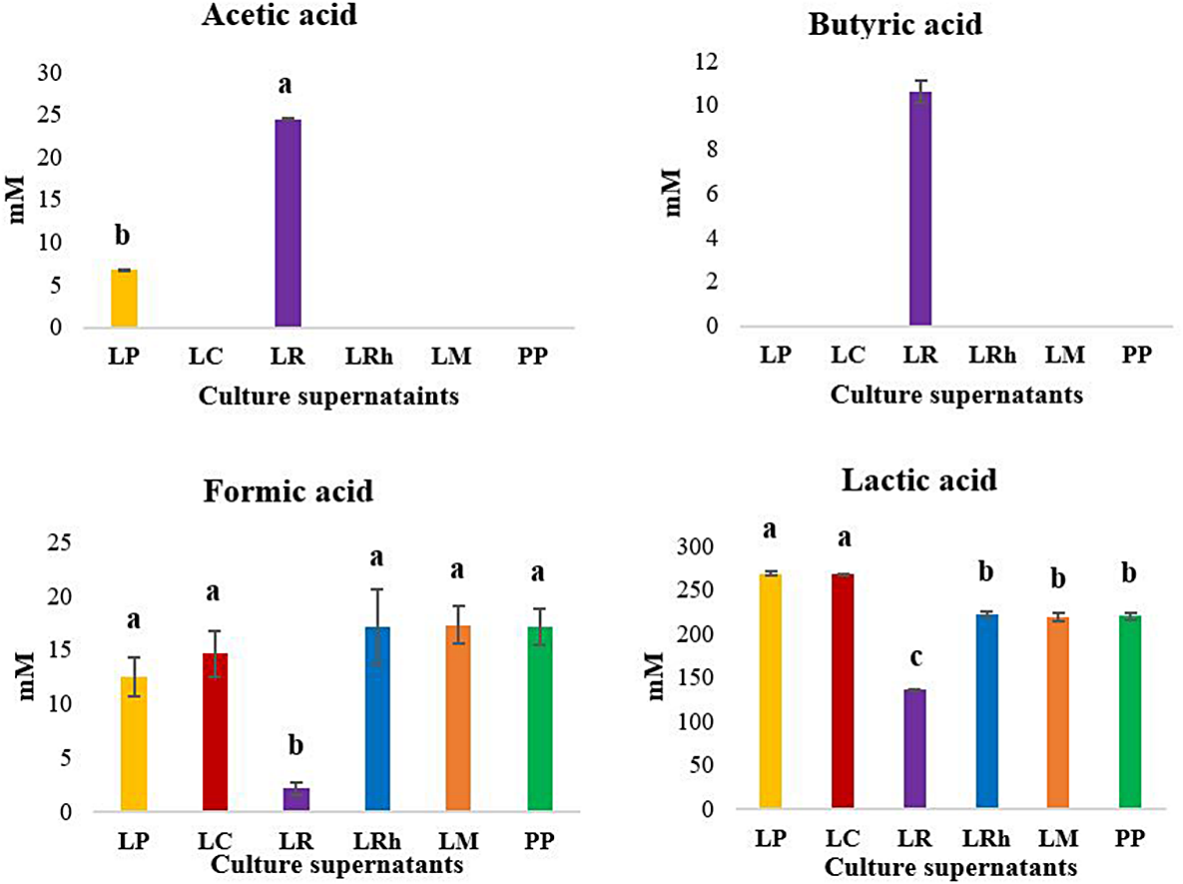
Organic acid quantification of the culture supernatants of LAB. **LP**: *L. plantarum*, **LC**: *L. casei*, **LR**: *L. reuteri*, **LRh**: *L. rhamnosus*, **LM**: *L. mesenteroides*, **PP**: *P. pentosaceus*. Error bars: ±SD. Homogenous means indicated by similar letters: Tukey’s HSD test (p value < 0.05)

The quantification of the organic acids in the culture supernatants is shown in Fig. 8. Propionic or citric acid production was not detected in any supernatants tested. There was significant acetic acid and butyric acid production in the *L. reuteri* while *L. plantarum* displayed a limited acetic acid production. Other LAB did not display significant production of these two organic acids. On the other hand, lactic acid and formic acid were found at high levels in the LAB strains which displayed highest anti-*Salmonella* and anti-*Campylobacter* properties. Limited inhibition of the pathogens was observed by *L. reuteri* while the least lactic acid and formic acid production was observed for the same strain. These results suggest a possible role for lactic and formic acids in the anti-*Salmonella* and anti-*Campylobacter* properties of the LAB studied.

## Discussion

Lactic acid bacteria (LAB) are a group of beneficial bacteria that have earned a reputation in inhibiting pathogens both *in-vitro* and *in-vivo* (Ibrahim et al. 2021). It is imperative to select a LAB strain which displays preferably a broad spectrum anti-pathogenic potential for applications to improve the gut health of livestock. The six LAB species that were assessed in the current study are used in multi-strain commercial probiotic supplements for poultry (JHJ Sp. z o.o. 2021) and this product displayed promising results in reduction of *Salmonella enteritidis* (Smialek et al. 2019) and *Campylobacter spp.* (Smialek et al. 2018) in broiler gastrointestinal tract (GIT). The current study evaluated the potential of individual LAB strains against three serovars of *Salmonella enterica* and *Campylobacter jejuni* in terms of bacteriostatic, bactericidal or co-aggregating properties along with their mode of action. *Lecuconostoc mesenteroides* has been identified as the most promising candidate LAB due to its anti-*Salmonella* and anti-*Campylobacter* activity. Moreover, the results of the current study demonstrated a significant role for lactic and formic acid production in this antimicrobial activity.

As the inhibition ability was lost when the culture supernatants of the strains used in the current study, were pH neutralized, the anti-*Salmonella* and anti-*Campylobacter* activity is likely to be associated with a pH effect. LAB are known to impart a pH lowering effect via producing different types of organic acids. Generally, the organic acids demonstrate a non-specific mode of action and thus a broad spectrum antimicrobial activity (Khan et al. 2022). The undissociated form of the organic acids are able to diffuse into the bacterial cells due to its lipophilic nature. Inside the cytoplasm, they dissociate to release H^+^ ions and reduce the intra-cytoplasmic pH of these pathogens. This eventually results in compromised metabolic functions accounted for bacteriostasis or bactericidal activity. Therefore, organic acids produced by LAB seems to be the likely cause for the strains observed inhibitory effects in the current study. Previous studies reported cases where the anti-pathogenic effects from different LAB strains were maintained (De Giani et al. 2019), decreased (Keeratikunakorn et al. 2023) and disappeared (Ołdak et al. 2020), when pH of the cell free supernatant was neutralized. These studies claim that when the antimicrobial activity is maintained, the inhibitory activity is not due to a pH/organic acid effect whereas decreased or no inhibitory activity is partially or completely due to the effects of pH/organic acid production, respectively. These claims are in agreement with our hypothesis that the inhibition observed by our LAB strains is likely to be due to organic acid production.

Further supporting this assumption, interestingly, different degrees of inhibition were observed for the cultures despite having similar pH. This possibly highlights the significance of specific organic acids produced by each LAB which may display different antimicrobial potential at the same pH. According to our results *L. reuteri* displayed almost similar pH to *L. mesenteroides* but displayed much less inhibition of all pathogens studied. It was clear that formic acid and lactic acid content were lowest in the culture supernatant of *L. reuteri* while *L. mesenteroides* displayed great production of these organic acids. Similarly, *L. reuteri* displayed higher production of acetic and butyric acids compared to other LAB studied. Burin et al. (2014) claimed that pathogen inhibition by acetic acid may be higher than lactic acid due to its lower dissociation ability compared to that of lactic acid. The current results suggest that the strains which produce greater amounts of both lactic acid and formic acid appear to cause more inhibition of pathogens compared to *L. reuteri* which produces acetic acid (which is more effective) but production is lower. It might also be possible that the observed antimicrobial properties are due to a synergistic effect of combinations of organic acids (produced by these LAB) as previously documented by Peh et al. (2020) against *Campylobacter* species. These authors observed a synergistic potential of caprylic acid, sorbic acid and caproic acid in inhibiting *Campylobacter jejuni* and *Campylobacter coli, in-vitro.*

LAB ferment sugars yielding mainly lactic acid to produce the energy necessary for their metabolism. Interestingly, LAB consist of diverse species belong to different genera including *Lactobacillus* (recently reclassified in to 25 genera such as *Lactiplantibacillus, Lacticaseibacillus, Limosilactobacillus, etc.*), *Leuconostoc, Pediococcus etc.* Although fermentation ability is a common feature of these bacteria, they are broadly divided into two major groups of fermenters namely, homofermentative and heterofermentative bacteria. The sole by-product of homofermentation is considered to be lactic acid while heterofermentation yields several by-products such as lactic acid, carbon dioxide (CO_2_), ethanol and/or acetic acid (Kim et al. 2022). Theoretically, the homofermenters produce 2 moles of lactic acid per 1 mol of glucose while heterofermenters produce less (1 mol) lactic acid per 1 mol of glucose (Kim et al. 2022). Therefore, it is indicative that these differences in fermentation metabolism may attribute to differences in organic acids and their quantities produced by the LAB in the current study. Interestingly, the six LAB were belonged to different fermentation groups. *P. pentosaceus* is considered more a homofermenter while the rest are obligate (*L. reuteri*) and facultative (*L. plantarum, L. casei. L. rhamnosus* and *L. mesenteroides*) heterofermenters. Therefore, another possibility that these strains displayed different degree of inhibition at the same pH, may be because of other metabolites produced such as ethanol or carbon dioxide production along with the organic acids.

Moreover, different strains from the same LAB species that were used in the current study, are known to produce bacteriocins such as Plantaricin which is produced by *L. plantarum* (Gong et al. 2010; Kumar et al. 2016), Pediocin by *P. pentosaceus* (Khorshidian et al. 2021), Caseicin by *L. casei* (Rammelsberg et al. 1990), Rhamnocin by *L. rhamnosus* (Jeong and Moon 2015), Mesenterocin from *L. mesenteroides* (Daba et al. 1991) etc. It is also important to highlight that certain LAB produce bacteriocins which lose their antimicrobial activity in neutral or alkaline pH conditions (Peng et al. 2023). Moreover, Keersmaecker et al. (2006) observed a non-proteinaceous broad spectrum antimicrobial compound which is synthesized by a *L. rhamnosus* strain and active under lower pH as mediated by accumulation of lactic acid. Therefore, apart from specific fermentation metabolites produced, it is also possible that these LAB displayed different degrees of inhibition due to the production of other proteinaceous or non-proteinaceous antimicrobial compounds which are only active under lower pH. Nevertheless, this theory must be confirmed by further investigation.

In the co-culture, not a single *Salmonella* colony forming unit (CFU) was observed at 24 h while considerable numbers of *Salmonella* were present after 10 h incubation. Nevertheless, the pH of both time points was similar. Thus, we suggest that although the pH was similar at both time points (by production of organic acids) it might take some time for the organic acids to diffuse into pathogenic cells, and interfere with the metabolic functions of *Salmonella* to completely eradicate them from the co-culture. However, another consideration is that these LABs produce a strong antimicrobial metabolite which can eradicate *Salmonella*, later in their exponential growth which might be activated at a lower pH as previously observed by Keersmaecker et al. (2006).

Moreover, the co-aggregation ability of a probiotic with a pathogen, is a good indication of *in-vivo* inhibition of pathogen colonization in the GIT. If a probiotic is able to co-aggregate with a pathogen, it is an advantage for the probiotic to release the antimicrobial compounds at a close proximity to these pathogenic bacteria preventing their colonization in the gut (Tuo et al. 2013). Therefore, *L. mesenteroides*, from our results displays the most promise to combat *Salmonella* Typhimuriumcolonization in the GIT of broiler chickens.

## Conclusion

Among the different strains of different genera belonging to lactic acid bacteria studied, *Leuconostoc mesenteroides* displayed the most significant overall anti-pathogenic properties against all the food borne pathogens used suggesting its potential for *in-vivo* applications to combat foodborne pathogens in broiler chickens.

## Data Availability

The datasets generated during and/or analyzed during the current study are available from the corresponding author on reasonable request.
